# Supplementary material for: Both major xanthophyll cycles present in nature promote nonphotochemical quenching in a model diatom
Source: Plant Physiol. 2025 Aug 23;199(1):kiaf371. doi: 10.1093/plphys/kiaf371 (PMC12449192; doi:10.1093/plphys/kiaf371)
Supplement: kiaf371_Supplementary_Data [file kiaf371_supplementary_data.zip › Supplementary material.pdf]

Supplementary Information S1. Detailed method for genetic transformation of *P. tricornutum*. A CRISPR-Cas9 site directed mutagenesis approach was used to knockout *ZEP2*, *ZEP3* and *VDE*. We applied a combination of high-resolution melting curve (HRM) analysis and Sanger sequencing to identify clones with disrupted native gene (Nymark et al., 2017). Induction-relaxation experiments were also performed in the preliminary screening phases to detect altered NPQ phenotypes. While *VDE* and *ZEP3* deficient lines showed a substantial lack of NPQ induction or recovery respectively, no clear phenotype alterations were observed for *ZEP2* deficient lines (**Supplementary Fig. S1**). Due to the diploid nature of *P. tricornutum* genome (Bowler et al., 2008), the presence of knockout mutations on both alleles was finally assessed with TOPO cloning (**Supplementary Fig. S2**). We selected clones that harbored indels of varying lengths resulting in a disrupted gene on both alleles (**Supplementary Fig. S3**). Following sequential rounds of singularization to obtain monoclonality and promote the loss of the episome, resulting lines were further confirmed with PCR genotyping (**Supplementary Fig. S4**). Full DNA sequences can be found in **Supplementary Table S5**.

With the site directed mutagenesis approach used in this study (Nymark et al., 2017), random mutations can be inserted in the targeted gene, which often results in small indels (Nymark et al., 2016) not traceable via gel electrophoresis (e.g., **Supplementary Fig. S2**). Moreover, compared to other approaches (i.e., homology-directed repair (HDR) (Bai et al., 2022) or random genomic integration via biolistic bombardment (Buck et al., 2019)) episomes are transiently maintained and do not stably integrate antibiotic resistance or transgenes into the host genome. This poses a disadvantage during screening but in turn prevents random integrations in the genome and allows the removal of the episome after mutant isolation, to reclaim antibiotic selection markers if required (e.g., for subsequent transformations) and to prevent off-target mutations.

After amplification of the targeted regions with TAQ (**Supplementary Fig. S2D**), we pre-selected ex-conjugants with high resolution melting curve analysis (HRM) (and chlorophyll *a* fluorescence phenotyping for *vde* and *zep3* mutants). Only a subset of clones showing clear divergence from the wt were further analyzed with

TOPO cloning to assess the presence of biallelic mutations. Only frame shifts resulting in premature stop codons or exceptionally large indels were considered as successful KOs. Alignments of final sequencing results of *zep2*, *zep3* and *vde* deficient lines and wt are shown in **Supplementary Fig. S2A-C**.

We further confirmed these results by PCR genotyping using HiDi polymerase, that selectively discriminates SNPs at the primers' 3′ end. We thus designed primers whose 3′ ends correspond to only one specific sequence (e.g., wt or KO) based on the original TOPO cloning results. We obtained a set of three primer pairs for each target gene: one specific for the native gene (e.g., *VDE*), and one for each allele of the corresponding deficient line (e.g., *vde KO* allele 1 and *vde KO* allele 2). In addition, we designed a primer pair that selectively amplified the newly introduced gene from the complemented line (e.g., *vde C*) based on the sequence of the complementation plasmid (one primer binding within the target gene and one primer binding a sequence of the pPTbsr backbone, absent in the wt). All primer sequences are reported in **Supplementary Table S2**.

With this approach we were able to reliably discriminate wt from edited alleles in mixed and monoclonal cell populations and thus could proof the absence of the native gene in each of the selected KO lines (**Supplementary Fig. S4**): native gene amplification (*VDE, ZEP2 and ZEP3*) gave a positive result only for wt DNA, while amplification of both alleles from the mutants was observed only in the corresponding DNA samples. As only exception, the PCR for *zep2 KO* allele 2 showed a positive amplification also in the wt sample (**Supplementary Fig. S4B)**; however, for this primer pair a size shift (corresponding to the known sequence for this allele) could be observed in *zep2 KO* compared to the wt, while both bands were amplified in the complemented line (*zep2 C*), confirming the genotyping of this allele despite the unpredicted amplification in the wt. Complementation lines were also successfully genotyped: all samples (*vde C*, *zep2 C* and *zep3 C*) showed a positive amplification for both KO alleles and for the primer pairs specific the

complemented line. As part of our quality control, PCR genotyping was repeated prior experiments to ensure absence of potential cross-contamination.

Alignments of the protein sequences deduced from *in silico* translation of the edited genes showed that both alleles of each mutant line had disrupted gene products that either resulted from out-of-frame shifts with premature stop codons or from large insertions/deletions (**Supplementary Fig. S3**). The full sequences of the target genes for wt, mutants (both alleles) and complemented lines are available as **Supplementary Table S5**. For all target genes we were able to isolate strains with different mutations on the two allele; thus, we did not observe the predominant loss of heterozygosity reported in previous studies (Bai et al., 2022; Nymark et al., 2016). This could be a result of selection or of differences in the transformation and mutagenesis approaches. However, we often observed a rearrangement of the allele-specific SNPs known in our wt type strain (annotated in **Supplementary Table S5**), which would be consistent with the high frequency of mitotic interhomolog recombination observed in *P. tricornutum* (Bulankova et al., 2021).

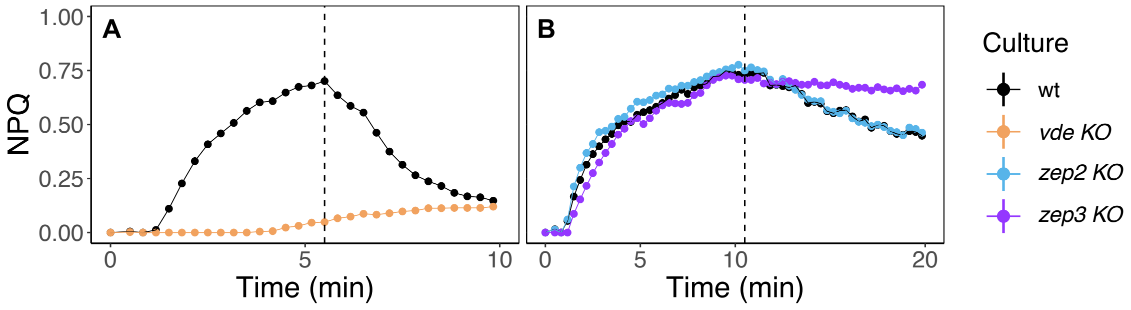

Supplementary Fig. S1. Preliminary induction-relaxation screening of *VDE*, *ZEP2* and *ZEP3* knockout lines and wt (representative example). The first pulse in the dark was followed (after 30 s) by light stress induction and recovery in low light. The switch from light stress to recovery is indicated by a dashed line. (*A*) screening of a *vde* deficient line (500m μmol photons m^{-2} s^{-1} for 5 min, followed by recovery at 28 μmol photons m^{-2} s^{-1}; n=1); (*B*) screening of *zep2* and *zep3* deficient lines (645m μmol photons m^{-2} s^{-1} for 10 min, followed by recovery at 28 μmol photons m^{-2} s^{-1}; n=1).

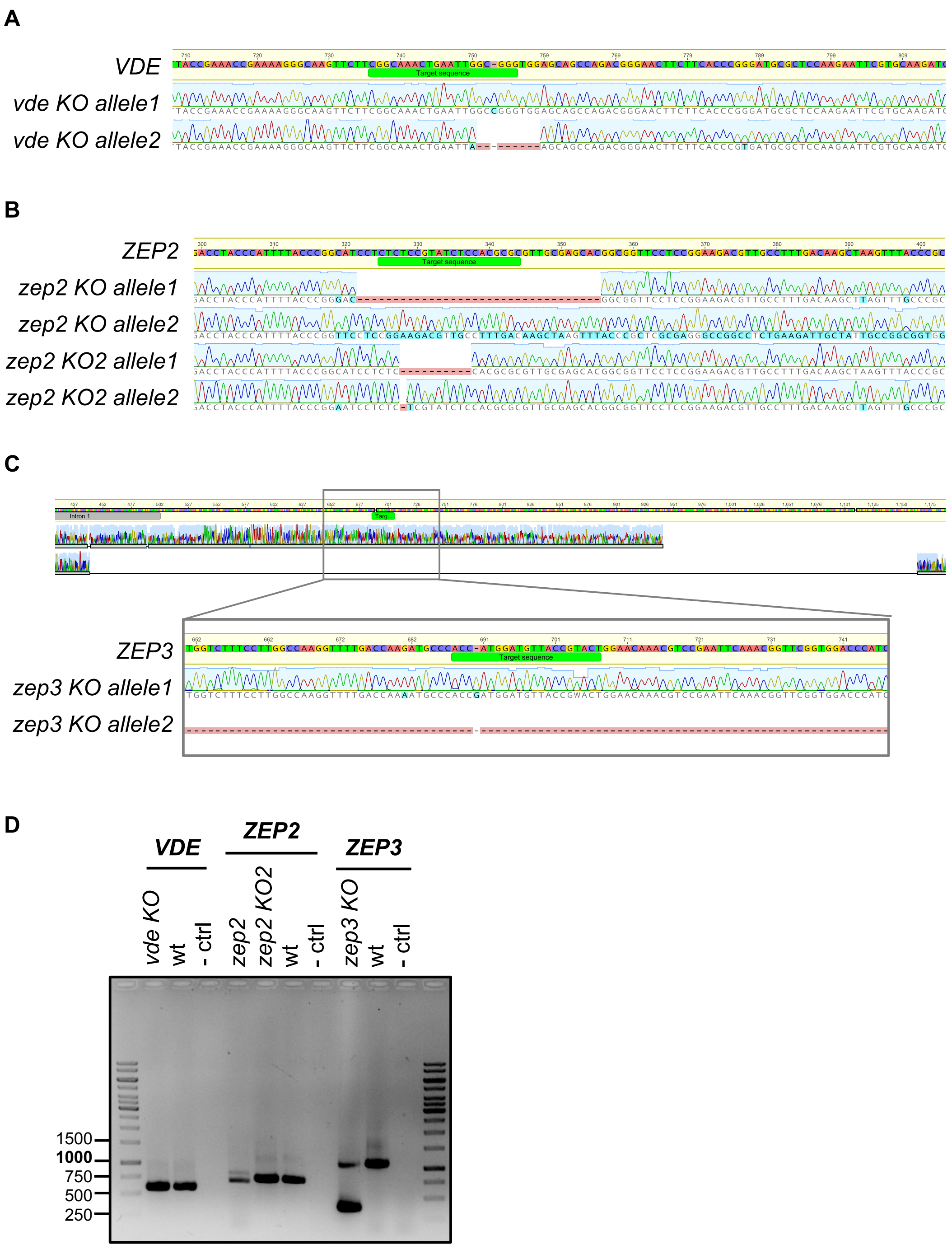

Supplementary Fig. S2. Partial DNA sequence alignments of both alleles of the respective target genes for all KO mutants generated in this study. Alignments were generated with Geneious 9.1 (Biomatters, New Zealand, 2016). Differences from wild type (*Pt*1; CCAP1055) are highlighted. (*A*) *vde KO*; (*B*) *zep2 KO* and *zep2 KO2*; (*C*) *zep3 KO* (*D*) gel electrophoresis of the corresponding TAQ

PCR products (1% TAE-agar stained with Roti GelStain) before TOPO cloning. Complete sequences are available as **Supplementary Table S5**.

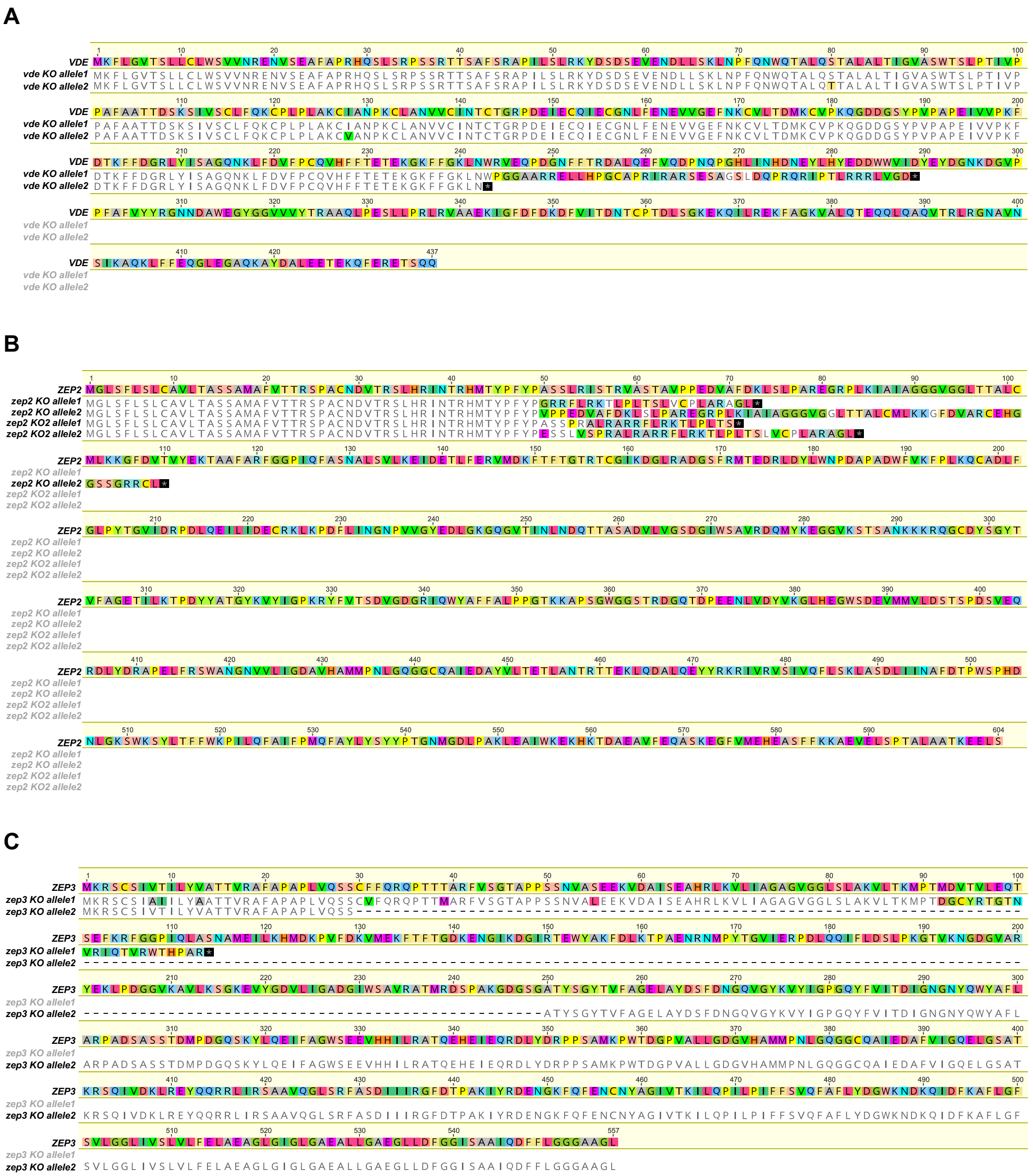

Supplementary Fig. S3. Complete protein sequence alignments of both allelic gene products for all knockout mutants generated in this study. Differences from wild type (*Pt*1; CCAP1055) are highlighted in the corresponding mutant sequence. (*A*) vde KO; (*B*) *zep2 KO* and *zep2 KO2*; (*C*) *zep3 KO*. Alignments were generated with Geneious 9.1 (Biomatters, New Zealand, 2016).

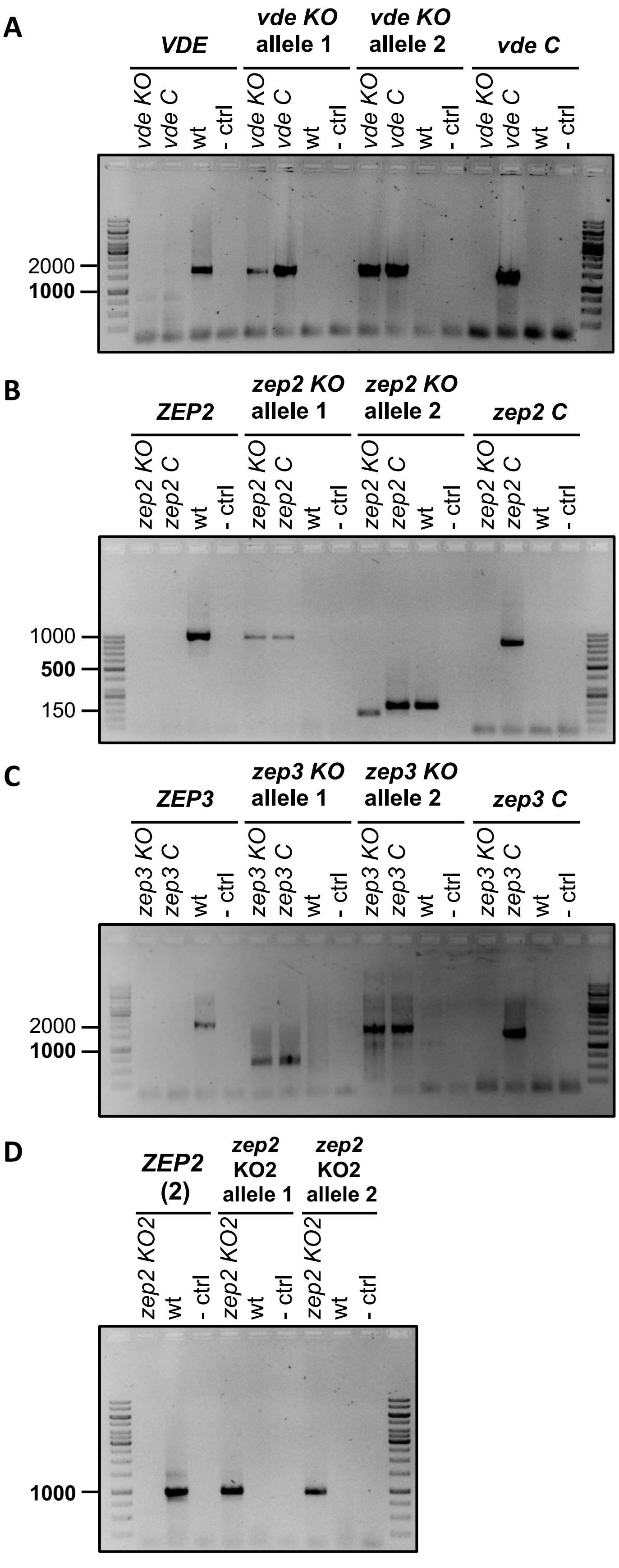
A
VDE
vde KO allele 1
vde KO allele 2
vde C
vde KO
vde C
wt
- ctrl
2000
1000
B
ZEP2
zep2 KO allele 1
zep2 KO allele 2
zep2 C
zep2 KO
zep2 C
wt
- ctrl
1000
500
150
C
ZEP3
zep3 KO allele 1
zep3 KO allele 2
zep3 C
zep3 KO
zep3 C
wt
- ctrl
2000
1000
D
ZEP2 (2)
zep2 KO2 allele 1
zep2 KO2 allele 2
zep2 KO2
wt
- ctrl
1000

Supplementary Fig. S4. Molecular characterization of transformed *P. tricornutum* mutants (KO and complementation lines) via PCR genotyping. Amplification from genomic DNA was performed according to HiDi manufacturer's instructions, using allele-specific primers binding specifically in each line (KO, wt and complemented). For KO lines, two different primer pairs were used to confirm the presence of both mutated alleles (allele 1 and allele 2). Corresponding primer sequences and additional information are available in **Supplementary Table S2** and **Supplementary Information S1**. For each gel, target sequences are indicated in **bold**, while sample names are placed vertically above each lane. Negative controls (- ctrl) were performed with nuclease-free water instead of DNA. (*A*) amplification of native *VDE*, *vde KO* allele 1, *vde KO* allele 2 and *vde C* specific sequences from *vde KO*, *vde C* and wt DNA samples; (*B*) amplification of native *ZEP2*, *zep2 KO* allele 1, *zep2 KO* allele 2 and *zep2 C* specific sequences from *zep2 KO*, *zep2 C* and wt DNA samples; (*C*) amplification of native *ZEP3*, *zep3 KO* allele 1, *zep3 KO* allele 2 and *zep3 C* specific sequences from *zep2 KO*, *zep3 C* and wt DNA samples; (*D*) amplification of native *ZEP2*, *zep2 KO2* allele 1 and *zep2 KO2* allele 2 specific sequences from *zep2 KO2* and wt DNA samples.

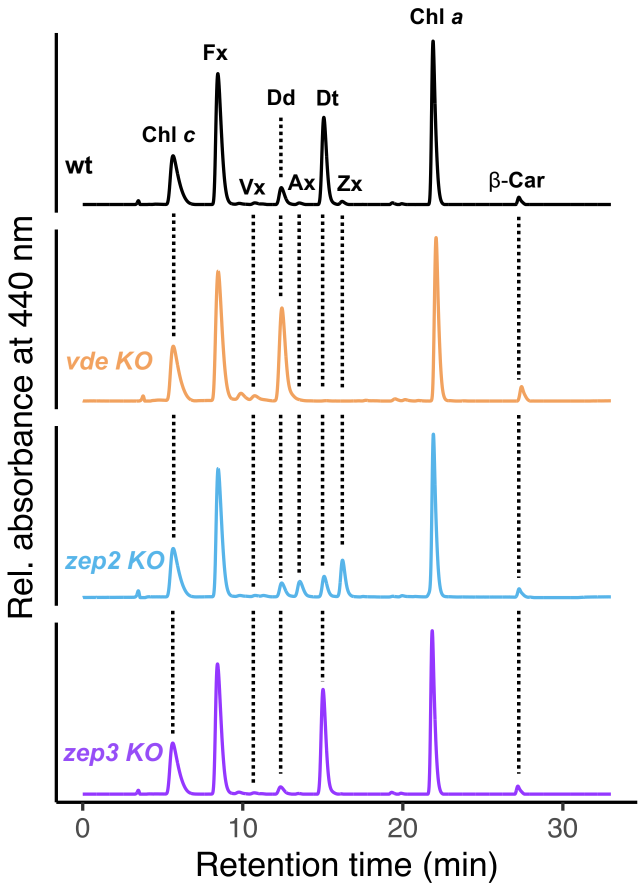

Supplementary Fig. S5. Representative chromatograms of pigment extracts from wt, *vde KO*, *zep2 KO* and *zep2 KO* after 6 h of HL. Chl c: chlorophyll *c*; Fx: fucoxanthin; Vx: violaxanthin; Dd: diadinoxanthin; Ax: antheraxanthin; Dt: diatoxanthin; Zx: zeaxanthin; Chl a: chlorophyll *a*; β-car: β,β-carotene**.**

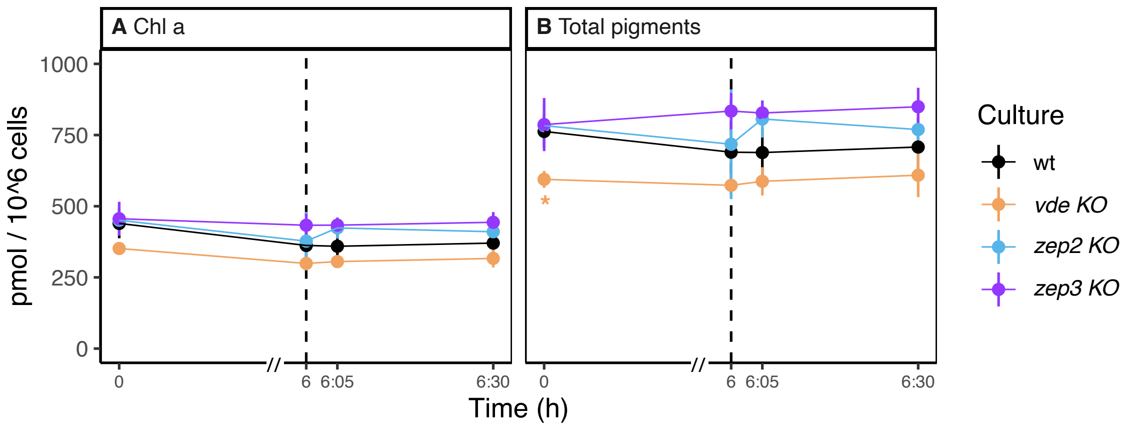

Supplementary Fig. S6. Content of chlorophyll *a* and total pigments in wt, *vde KO*, *zep2 KO* and *zep3 KO* after 5 days under HL:LL regime. Pigment content is expressed as a cell count ratio (pmol/10^6 cells) (average ± sd, n=3). Time 0 is defined as the beginning of the HL phase (i.e. the sampling point in LL that immediately precedes the onset of HL on the fifth day of HL:LL treatment). The 6 h of HL phase and 30 min of recovery phase (LL) are separated with a dashed line. On the x-axis, recovery phase (from 6 to 6:30 h) was artificially enlarged to allow better visualization; axis break is indicated by a double dash (//). (*A*) chlorophyll *a* (Chl *a*); (*B*) total pigments. Statistical significance marks indicate significant differences between the corresponding mutant line and wt at each time point, according to adjusted p-value of multiple comparison t-test (*: $p < 0.05$).

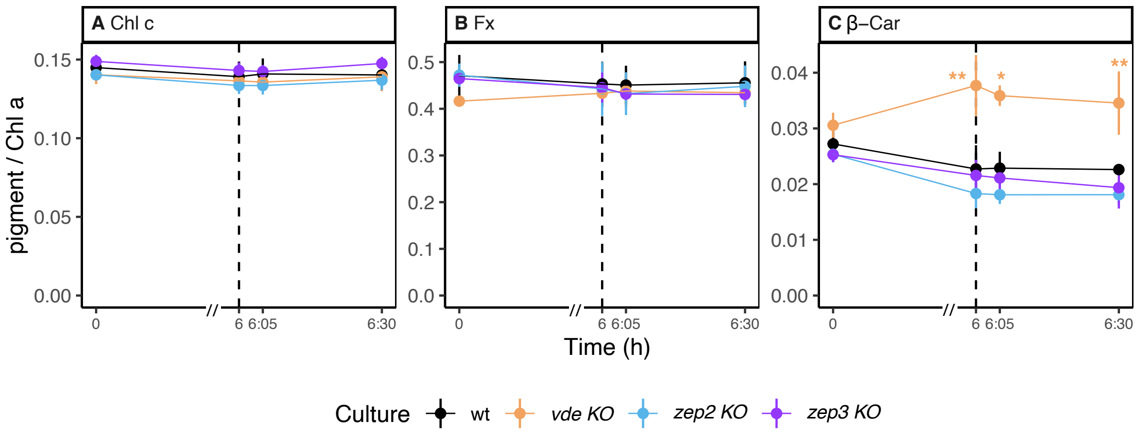

Supplementary Fig. S7. Content of other photosynthetic pigments and carotenes in wt, *vde KO*, *zep2 KO* and *zep3 KO* after 5 days under HL:LL regime. Pigment content is expressed as pigment per chlorophyll *a* (mol/mol) (average ± sd, n=3). Time 0 is defined as the beginning of the HL phase (i.e. the sampling point in LL that immediately precedes the onset of HL on the fifth day of HL:LL treatment). The 6 h of HL phase and 30 min of recovery phase (LL) are separated with a dashed line. On the x-axis, recovery phase (from 6 to 6:30 h) was artificially enlarged to allow better visualization; axis break is indicated by a double dash (//). (*A*) chlorophyll *c* (Chl *c*); (*B*) fucoxanthin (Fx); (*C*) β,β-carotene (β-Car). Statistical significance marks indicate significant differences between the corresponding mutant line and wt at each time point, according to adjusted p-value of multiple comparison t-test (*: $p < 0.05$; **: $p < 0.005$).

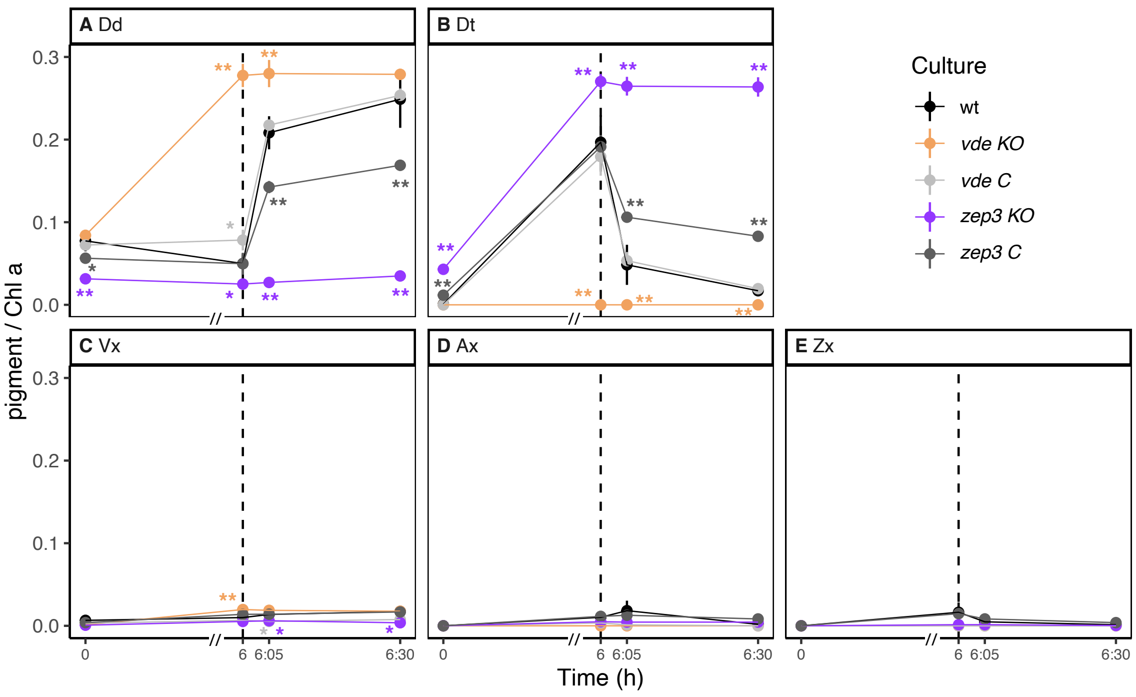

Supplementary Fig. S8. Content of Dd and Vx cycle pigments in *vde KO* and *zep3 KO*, their respective complementation lines (*vde C*, *zep3 C*) and wt after 5 days under HL:LL regime. Pigment content is expressed as pigment per chlorophyll *a* (mol/mol) (average ± sd, n=3). Time 0 is defined as the beginning of the HL phase (i.e. the sampling point in LL that immediately precedes the onset of HL on the fifth day of HL:LL treatment). The 6 h of HL phase and 30 min of recovery phase (LL) are separated with a dashed line. On the x-axis, recovery phase (from 6 to 6:30 h) was artificially enlarged to allow better visualization; axis break is indicated by a double dash (//). (*A*) diadinoxanthin (Dd); (*B*) diatoxanthin (Dt); (*C*) violaxanthin (Vx); (*D*) antheraxanthin (Ax); (*E*) zeaxanthin (Zx). Statistical significance marks indicate significant differences between the corresponding mutant line and wt at each time point, according to adjusted p-value of multiple comparison t-test (*: $p < 0.05$; **: $p < 0.005$).

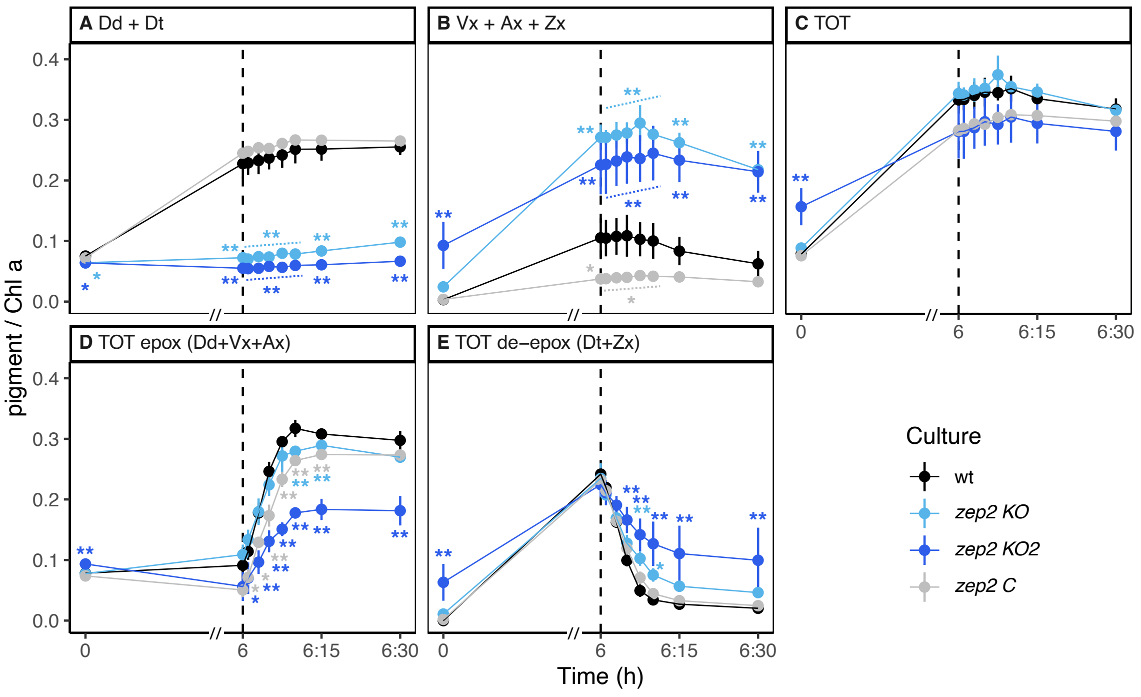

Supplementary Fig. S9. Total pools of Dd and Vx cycle pigments in wt, *zep2 KO*, *zep2 KO2* and *zep2* C after 5 days under HL:LL regime. Pigment content is expressed as pigment per chlorophyll *a* (mol/mol) (average ± sd, n=3). Time 0 is defined as the beginning of the HL phase (i.e. the sampling point in LL that immediately precedes the onset of HL on the fifth day of HL:LL treatment). The 6 h of HL phase and 30 min of recovery phase (LL) are separated with a dashed line. On the x-axis, recovery phase (from 6 to 6:30 h) was artificially enlarged to allow better visualization; axis break is indicated by a double dash (//). (*A*) total pool of Dd cycle pigments (Dd + Dt); (*B*) total pool of Vx cycle pigments (Vx + Ax + Zx); (*C*) sum of all Dd and Vx cycle pigments; (*D*) total pool of epoxidized pigments across different xanthophyll cycles (Dd + Vx + Ax); (*E*) total pool of de-epoxidized pigments across different xanthophyll cycles (Dt + Zx). Statistical significance marks indicate significant differences between the corresponding mutant line and wt at each time point, according to adjusted p-value of multiple comparison t-test (*: $p < 0.05$; **: $p < 0.005$).

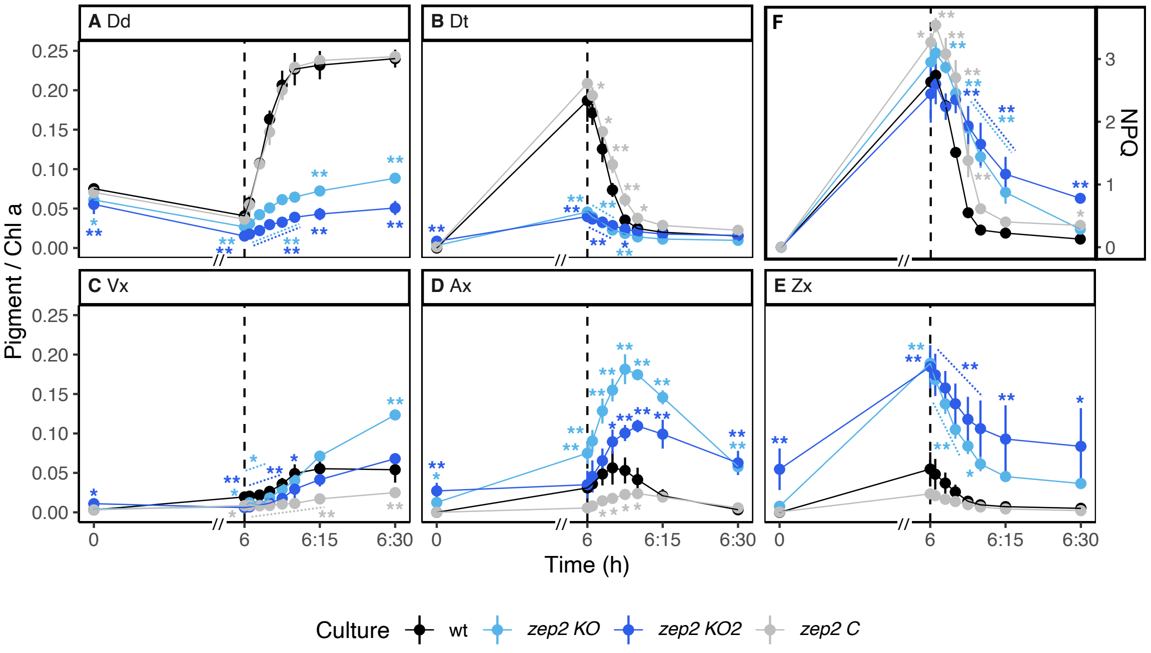

Supplementary Fig. S10. Content of Dd and Vx cycle pigments coupled with NPQ analysis in wt, *zep2 KO*, *zep2 KO2* and *zep2 C* after 5 days under HL:LL regime. Pigment content is expressed as pigment:chlorophyll *a* ratio (mol/mol) (average ± sd, n=3). Time 0 is defined as the beginning of the HL phase (i.e. the sampling point in LL that immediately precedes the onset of HL on the fifth day of HL:LL treatment). The 6 h of HL phase and 30 min of recovery phase (LL) are separated with a dashed line. On the x-axis, recovery phase (from 6 to 6:30 h) was artificially enlarged to allow better visualization; axis break is indicated by a double dash (//). (*A*) diadinoxanthin (Dd); (*B*) diatoxanthin (Dt); (*C*) violaxanthin (Vx); (*D*) antheraxanthin (Ax); (*E*) zeaxanthin (Zx); (*F*) NPQ. Statistical significance marks indicate significant differences between the corresponding mutant line and wt at each time point, according to adjusted p-value of multiple comparison t-test (*: $p < 0.05$; **: $p < 0.005$).

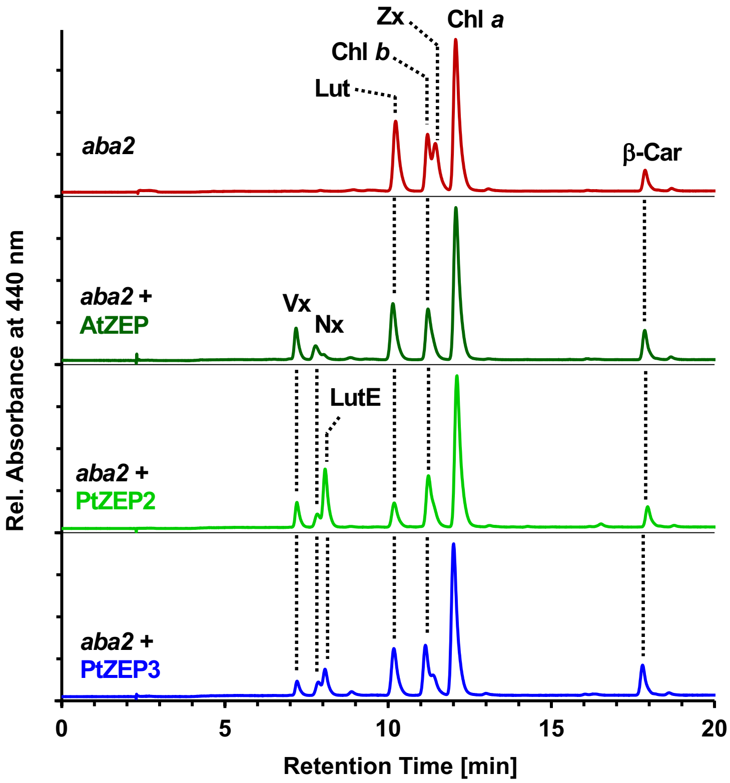

Supplementary Fig. S11. Transient expression of *P. tricornutum* *ZEP2* or *ZEP3* in leaves of the ZEP-deficient *Nicotiana plumbaginifolia aba2* mutant resulted in the formation of violaxanthin (Vx), its derivative neoxanthin (Nx), and lutein epoxide (LutE). Transient expression of the *ZEP* from Arabidopsis thaliana (AtZEP) as control yielded only Vx and Nx. Other pigments: β,β-carotene (β-Car); chlorophyll (Chl); lutein (Lut); zeaxanthin (Zx).

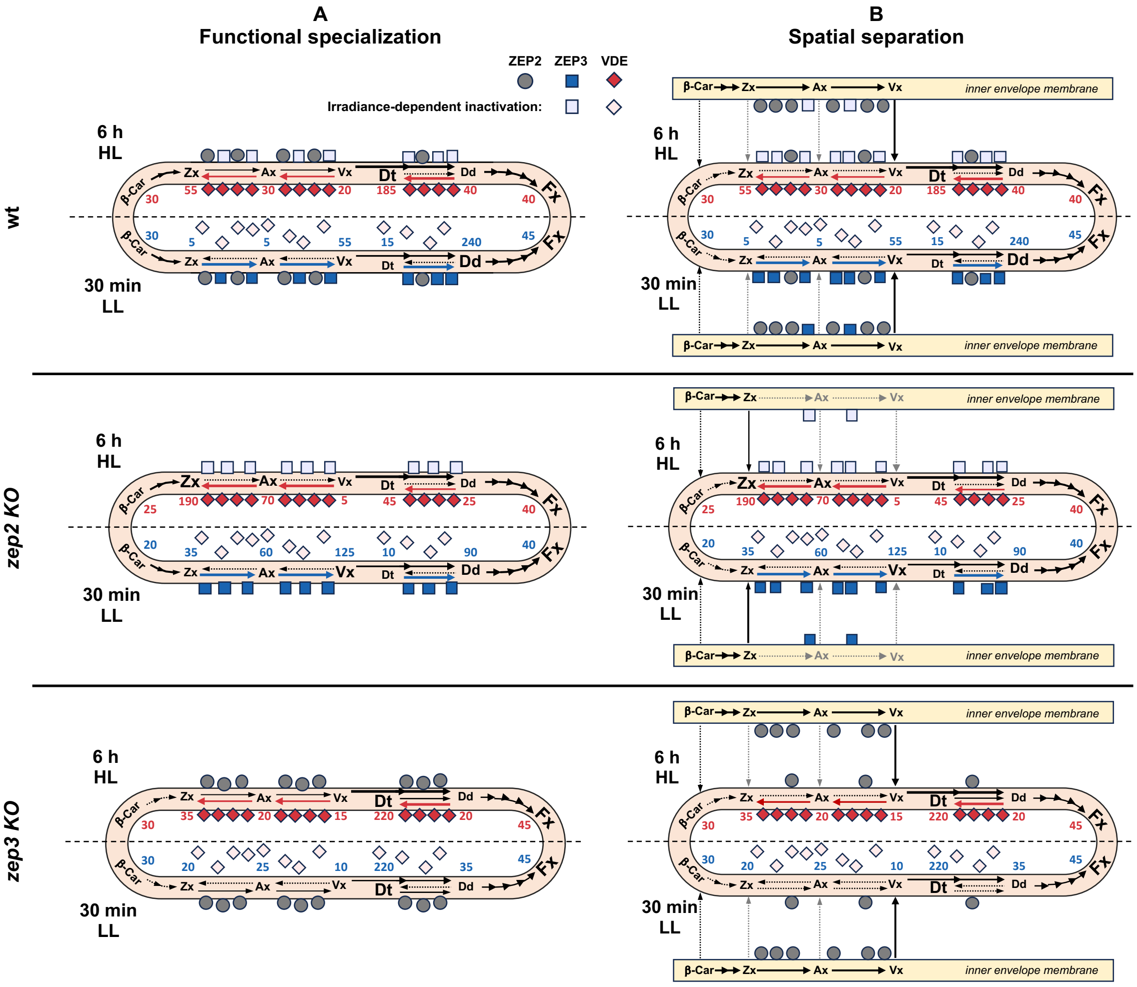

Supplementary Fig. S12. Detailed models explaining the observed differences in xanthophyll accumulation between *zep2 KO*, *zep3 KO* and wt. Different panels show the state of each line at 6h of HL treatment (top) and after 30 min of recovery under LL (bottom). Colored arrows show preferential direction of xanthophyll cycles under HL (red) or LL (blue), dashed arrows represent minor or alternative flows, numbers represent the relative concentration of each pigment at the corresponding time point (mol xanthophyll:10^3 mol chlorophyll *a*, approximated average from the second experiment, n=3). (*A*) Selective accumulation of Vx/Ax/Zx in *zep2 KO* explained by functional differences in the activity of ZEP2 and ZEP3. In this scenario, ZEP3 displays the properties previously attributed to the ZEP of diatoms, i.e., it is selectively inactivated by HL, being active almost exclusively under non-saturating irradiances to rapidly tune

down NPQ by converting Dt into Dd (Blommaert et al., 2021; Goss et al., 2006a), while ZEP2 has properties closer to the ZEP of higher plants and green algae, i.e., a constant activity under any irradiance (including darkness and HL) (Gilmore et al., 1994; Goss et al., 2006b, 1998; Siefermann and Yamamoto, 1975). Lack of ZEP2 causes the accumulation of Zx/Ax in *zep2 KO* and consequent reduction of Dd/Dt synthesis under HL due to lack of epoxidation activity to counteract VDE. Conversely, absence of ZEP3 determines lack of rapid Dt epoxidation in *zep3 KO* during LL recovery, while ZEP2 sustains the *de novo* formation of Vx and Dd under HL, albeit being unable to rapidly epoxidize Dt under LL recovery (at least within a time frame of 30 min investigated in this study). (*B*) The two *zep* phenotypes as result of the different localization of ZEP2 and ZEP3 in the chloroplast. In this model, ZEP2 is mostly located at the inner envelope, where it is responsible for the *de novo* synthesis of Vx from Zx; Vx is then transported to the thylakoid membranes for further conversion to Dd and fucoxanthin. In absence of ZEP2, carotenoid biosynthesis in the envelopes already ceases at the level of Zx, which is translocated instead of Vx to the thylakoid membranes. ZEP3 is confined mainly at the thylakoid membranes where it drives xanthophyll cycle-related Dt epoxidation, that results substantially inhibited in absence of this isoform. In this model ZEP2 displays a considerably higher epoxidase activity in the envelope than the experimentally determined epoxidation rates of Dt and Zx located in the thylakoid membranes.

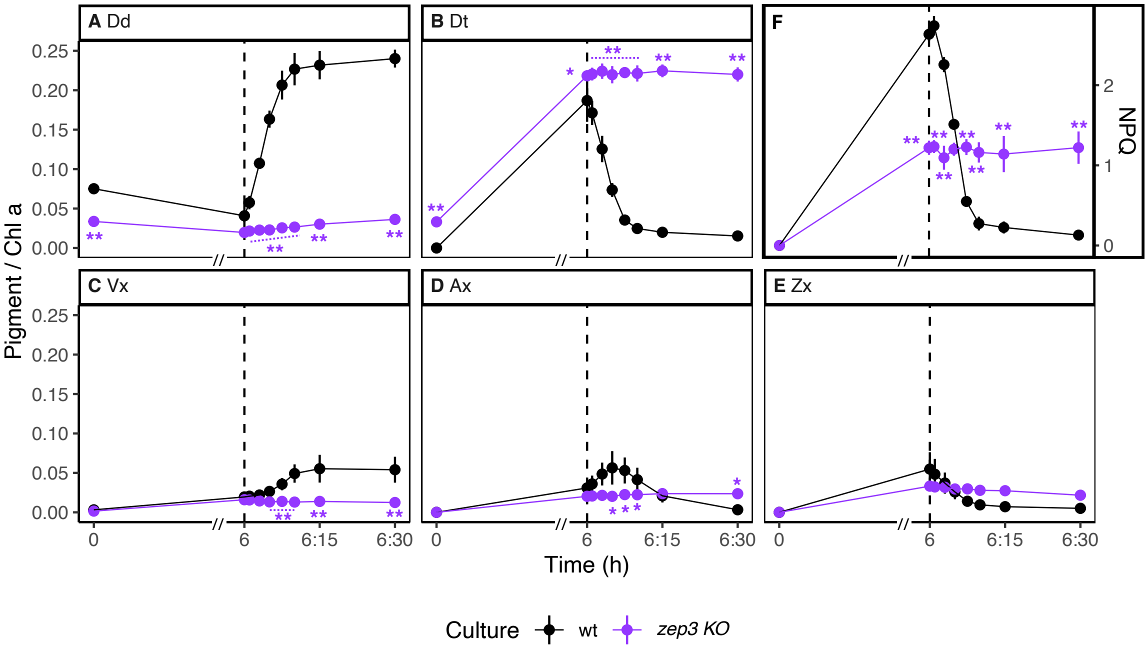

Supplementary Fig. S13: Content of Dd and Vx cycle pigments coupled with NPQ analysis in wt and *zep3 KO* after 5 days under HL:LL regime. Pigment content is expressed as pigment:chlorophyll *a* ratio (mol/mol) (average ± sd, n=3). Time 0 is defined as the beginning of the HL phase (i.e. the sampling point in LL that immediately precedes the onset of HL on the fifth day of HL:LL treatment). The 6 h of HL phase and 30 min of recovery phase (LL) are separated with a dashed line. On the x-axis, recovery phase (from 6 to 6:30 h) was artificially enlarged to allow better visualization; axis break is indicated by a double dash (//). (*A*) diadinoxanthin (Dd); (*B*) diatoxanthin (Dt); (*C*) violaxanthin (Vx); (*D*) antheraxanthin (Ax); (*E*) zeaxanthin (Zx); (*F*) NPQ; due to the substantial lack of Dt recovery in this mutant (as shown by the presence of Dt at time 0 and 6:30 h), the calculated NPQ depicted in this figure is most likely underestimated (measured Fm used to calculate NPQ is most likely lower compared to the real maximum fluorescence that would be measured in a fully relaxed state). Statistical significance marks indicate significant differences between the corresponding mutant line and wt at each time point, according to adjusted p-value of multiple comparison t-test (*: $p < 0.05$; **: $p < 0.005$).

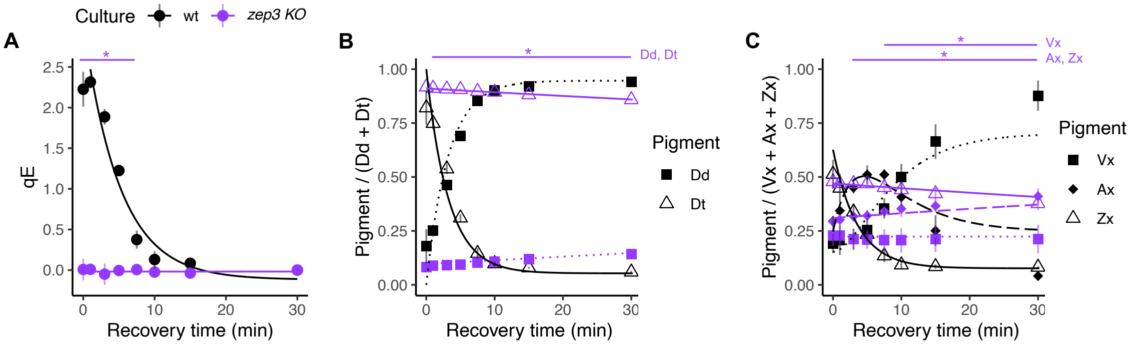

D

	qE	Dt → Dd	Zx → Ax	Ax → Vx	Ratios					
	R0	k_T [min^{-1}]	k_Z [min^{-1}]	k_A [min^{-1}]	k_T/k_Z	k_Z/k_T	k_T/k_A	k_A/k_T	k_Z/k_A	k_A/k_Z
**wt**	- 0.197 ± 0.025	0.310 ± 0.017	0.280 ± 0.035	0.167 ± 0.076	1.11 ± 0.08	0.90 ± 0.06	2.10 ± 0.81	0.53 ± 0.22	1.87 ± 0.61	0.58 ± 0.22
zep3 KO	NA NA	0.002 ± 0	0.005 ± 0	0.001 ± 0	0.4 ± 0	2.5 ± 0	3.33 ± 1.15	0.33 ± 0.14	8.33 ± 2.88	0.13 ± 0.06

Supplementary Fig. S14. First-order kinetics of qE recovery and of re-epoxidation of xanthophyll cycle pigments in wt and *zep3 KO* during 30 min of recovery phase after HL exposure, following 5 days under HL:LL regime. Data are expressed as average ± sd (n=3). (*A*) recovery rates of qE; (*B*) epoxidation of diadinoxanthin (Dd) to diatoxanthin (Dt); (*C*) epoxidation of zeaxanthin (Zx) via antheraxanthin (Ax) to violaxanthin (Vx); (*D*) corresponding rate constants of: qE recovery (R0); Dt to Dd epoxidation (k_T); Zx to Ax epoxidation (k_Z); Ax to Vx epoxidation (k_A). Bars with statistical significance marks (*) on top indicate time points showing significant differences between *zep3 KO* and wt, according to adjusted p-value of multiple comparison t-test ($p < 0.05$). For *zep3 KO*, no R0 values were determined as qE did not show significant changes during recovery. Due to the substantial lack of Dt recovery in this mutant (also shown in Supplementary Fig. S11), the calculated qE is artificially always close to 0 (Fm'' measured at 30 min of recovery and used to calculate qE is almost equal to the Fm' of other time points sampled between 6 h of light stress and 30 min of recovery due to substantial lack of recovery). This doesn't mean that this mutant is not performing qE, but rather that this cannot be appropriately calculated due to substantial lack of recovery.

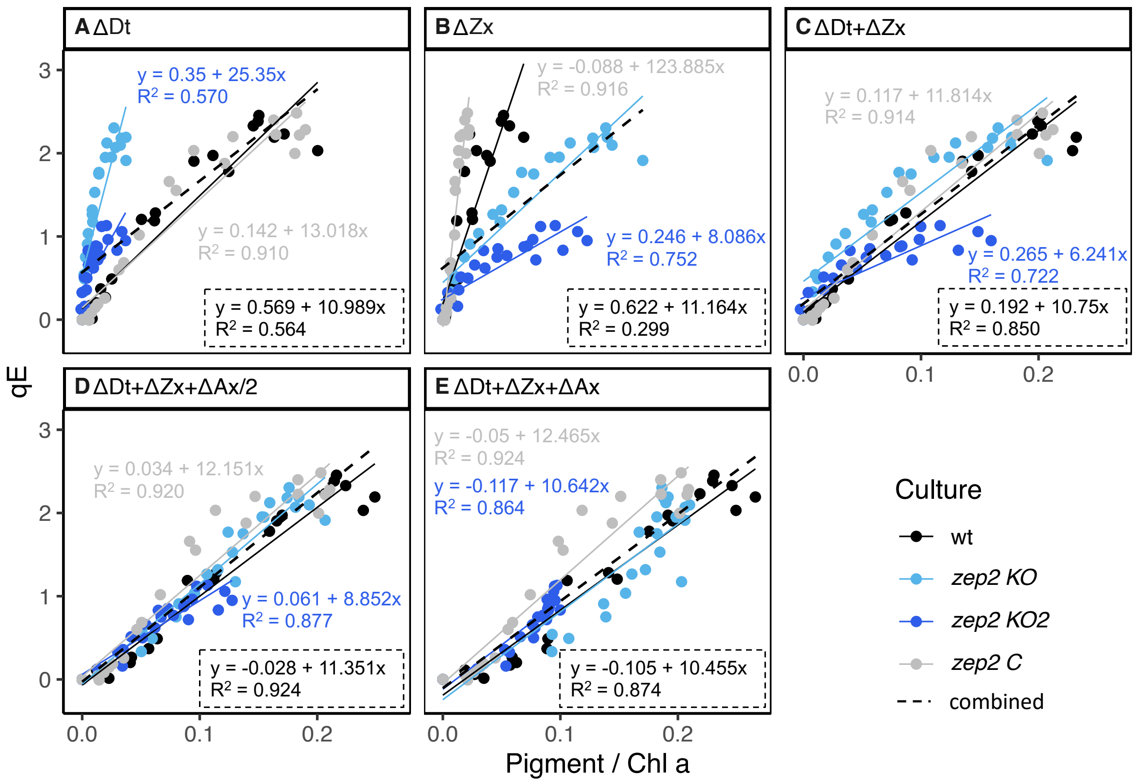

Supplementary Fig. S15. Correlation between qE and de-epoxidized pigments in *zep2 KO, zep2 KO2* and *zep2 C* and wt during recovery from 6 h of HL exposure, following 5 days under HL:LL regime. Each point represents a separate measurement, repeated for 3 independent biological replicates. To exclude the effect of photoinhibition (qI), qE and pigment content (Δp) were calculated as qE = Fm''-Fm'/Fm' and Δp = [p]-[p]'', where Fm'' and [p]'' represent the corresponding values at the end of the recovery phase (30 min). For each strain, different calculations of de-epoxidized pigment pool are displayed, with corresponding linear models and R^2. Equations and R^2 for wt and *zep2 KO* are reported in **Fig. 6**. Dashed lines represent the linear model obtained when considering all points from different lines as one unique population (combined); corresponding R^2 is displayed in the dashed text box at the bottom right corner of each facet. (*A*) diatoxanthin (Dt); (*B*) zeaxanthin (Zx); (*C*) sum of Dt and Zx; (*D*) sum of Dt, Zx and ½ antheraxanthin (Ax); (*E*) sum of Dt, Zx and Ax.

A

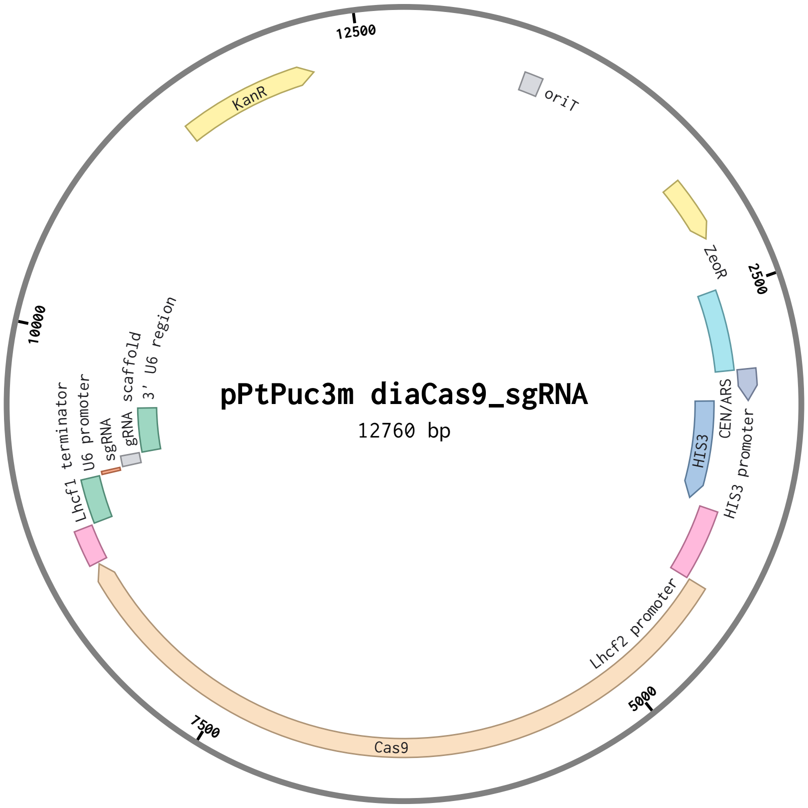
pPtPuc3m diaCas9_sgRNA
12760 bp
12500
KanR
oriT
ZeoR
2500
10000
CEN/ARS
HIS3
HIS3 promoter
Lhcf1 terminator
U6 promoter
sgRNA
gRNA scaffold
3' U6 region
Lhcf2 promoter
Cas9
5000
7500

B

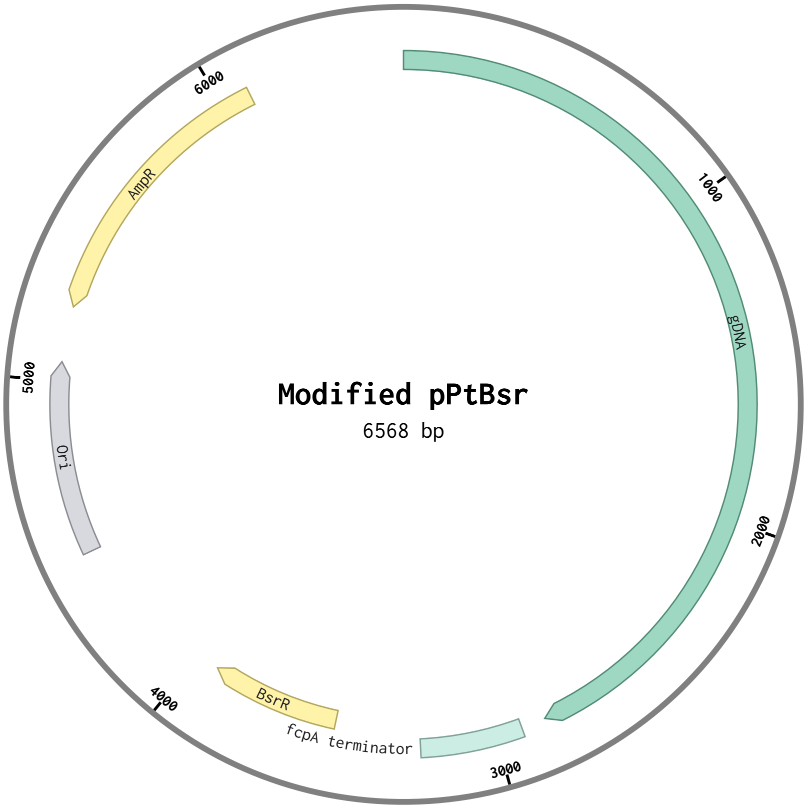
Modified pPtBsr
6568 bp
6000
1000
AmpR
gDNA
5000
Ori
2000
4000
BsrR
fcpA terminator
3000

Supplementary Fig. S16. Vector maps. (*A*) pPtPuc3m diaCas9_sgRNA vector for CRISPR/Cas9 gene knockout. Key elements are highlighted: diaCas9 (orange) under regulation of Lhcf2 promoter and Lhcf1 terminator (pink); sgRNA (red) with gRNA scaffold (grey) regulated by U6 promoter and followed by U6 3' target region (green); zeocin (Zeo) and kanamycin (Kan) resistance cassettes (yellow); origin of transfer (oriT; gray); CEN/ARS (light blue) followed by HIS3 promoter and HIS3 (blue). The full plasmid sequence and additional information are provided at AddGene (Plasmid #109219) and in Sharma et al. (2018). (*B*) Modified pPTbsr vector for complementation. For each target gene, we removed the original fcpA promoter and added the whole length wt gene including the predicted endogenous promoter and terminator (gDNA; dark green) within the original multiple cloning site (MCS). All other components were unchanged from the original plasmid. Key elements are highlighted: fcpA terminator (light green); origin of replication (Ori; gray); Blasticidin (Bsr) and Ampicillin (Amp) resistance cassettes (yellow). The full plasmid sequence and additional information are provided at AddGene (Plasmid #117696) and in Buck et al. (2018).

Supplementary Table S1. sgRNA sequences used for CRISPR/Cas9 genome editing.

sgRNA	Target gene
GCGCGTGGAGATACGGAGAG	*ZEP2*
AGTACGGTAACATCCATGGT	*ZEP3*
CGGCAAACTGAATTGGCGGG	*VDE*

Supplementary Table S2. Primer sequences used for mutant screening and PCR genotyping.

Mix name	Binding target	Primer F (5′-3′)	Primer R (5′-3′)	Size (bp)	T ann. (°C)
**PCR screening**					
VDE	*VDE*	VDE_longF: GTTCGATGTATTTCCCTGCC	VDE_longR: GCACCTTCTAGACCTTGCTC	594	58
ZEP2	*ZEP2*	ZEP2_longF: GGAATCCTTCTGTCACTAAC	ZEP2_longR: TGAAAGGTAAGTTTTCGTGT	752	45
ZEP3	*ZEP3*	ZEP3_longF: ATACGTAGCCACCACGGTGA	ZEP3_longR: GAAAACCGTGTACCCCGAGT	1106	56
**HRM**					
VDE	*VDE*	VDE_longF	VDE_shortR: CGGCTGATTCGGATCTTG	151	
ZEP2	*ZEP2*	ZEP2_shortF: CATAGAATCAATACAAGGCACAT	ZEP2_shortR: CGTGCTCGCAACGCGCGT	111	
ZEP3	*ZEP3*	ZEP3_shortF: CCGGCTCAAAGTGCTGATTG	ZEP3_shortR: GCGTTACTAGCGAGCTGGAT	150	
**PCR genotyping**					
VDE	wt *VDE*	VDE_1: GGCAAACTGAATTGGCG	VDE_A: TGGCTGGAAAAATTGCGTCC	1791	58
vde KO allele 1	*vde KO* (allele 1)	VDE_2: GGCAAACTGAATTGGCC	VDE_A	1791	58
vde KO allele 2	*vde KO* (allele 2)	VDE_3: TCTTCGGCAAACTGAATTAA	VDE_A	1787	58
vde C	*vde C*	VDE_1	pPT_F: GTGACACTATAGAACCAGATCCC	1409	58
ZEP2	wt ZEP2 (variant for *zep2 KO* genotyping)	ZEP2_A: GACAAAACCTTGGCGAGCTG	ZEP2_1: CGCGCGTGGAGATACGG	987	60
zep2 KO allele 1	*zep2 KO* (allele 1)	ZEP2_A	ZEP2_2: CGGAGGAACCGCCGTC	968	60
zep2 KO allele 2	*zep2 KO* (allele 2)	ZEP2_B: GACCTACCCATTTTACCCGGT	ZEP2_3: TCAACATGCAGAGAGCGGTC	140	60
zep2 C	*zep2 C*	pPT_F	ZEP2_1	812	58
ZEP3	wt ZEP3	ZEP3_A: TTGGGTACGACGATTTCG	ZEP3_1: CCAGTACGGTAACATCCATG	1948	60
zep3 KO allele 1	*zep3 KO* (allele 1)	ZEP3_longF	ZEP3_2: CCAGTACGGTAACATCCATC	624	60
zep3 KO allele 2	*zep3 KO* (allele 2)	ZEP3_A	ZEP3_3: CCCGAGTAGGTGGCGG	1693	60
zep3 C	*zep3 C*	pPT_F	ZEP3_4: CGGACGTTTGTTCCAAAACC	1374	60
ZEP2 (2)	wt ZEP2 (variant for *zep2 KO2* genotyping)	ZEP2_A	ZEP2_1b: GCGCGTGGAGATACGGA	986	60
zep2 KO2 allele 1	*zep2 KO2* (allele 1)	ZEP2_A	ZEP2_4: CAACGCGCGTGGAGAG	980	58
zep2 KO2 allele 2	*zep2 KO2* (allele 2)	ZEP2_A	ZEP2_5: CAACGCGCGTGGAGATACGA	989	58

Supplementary Table S3. Primer sequences used for the generation and sequencing of complementation plasmids.

Sequence	Target(s)
TTTCTAATCACGATCGACCTGG	Amplification of *VDE* whole length (F)
GCACCGACCCAAAATCAAGAG	Amplification of *VDE* whole length (R)
AATGCGCTTTCGAACGGTG	Amplification of *ZEP2* whole length (F)
ACGTCGGCAACGCGAAG	Amplification of *ZEP2* whole length (R)
GGTAACCTTTCCTGGCCTTTCC	Amplification of *ZEP3* whole length (F)
AGCCTCTACACGTATGTGTTTCG	Amplification of *ZEP3* whole length (R)
GTGACACTATAGAACCAGATCCC	Sequencing of gene insertion site (F), **pPT_F**
TTAAGGAAGGATAGAGACT	Sequencing of gene insertion site (R)
CTCTCGGGCTCCAATTTTG	*VDE* intermediate sequencing (1)
GTTCGATGTATTTCCCTGCC	*VDE* intermediate sequencing (2), **VDE_longF**
CGGCTGATTCGGATCTTG	*VDE* intermediate sequencing (3), **VDE_shortR**
GCACCTTCTAGACCTTGCTC	*VDE* intermediate sequencing (4), **VDE_longR**
ATTTTACCCGGCAAGTAGC	*ZEP2* intermediate sequencing
ATGAAAAGATCTTGCAGTAT	*ZEP3* intermediate sequencing (1)
AGACAACCCACTACAACGGC	*ZEP3* intermediate sequencing (2)
TCTATGACACCCGTATAC	*ZEP3* intermediate sequencing (3)
GCTGAACCGAGCTCTTGTCC	*ZEP3* intermediate sequencing (4)

Given the length of all target genes (*VDE*, *ZEP2*, *ZEP3*) several primers were used for sequencing of the complete gene inserted in the pPTbsr vector. Names of primers that were used also for mutant screening and genotyping (**Supplementary Table S2**) are indicated in bold.

Supplementary Table S4. Pigment content of different mutants and wt under pre-experimental conditions (LL control) and after 5 days under HL:LL regime, before the onset of HL.

	Chl c	Fx	Fx-i	Vx	Dd	Ax	Dt	Zx	Chl a	β-car	c-β-car	TOT
rt (min)	5.5	8	9.5	10.5	12	13	14.5	15.5	21.5	27	27.5	
λ_{max} (nm)	337<br>445<br>632	448	441	418<br>441<br>470	447<br>477	447<br>475	453<br>481	453<br>479	414<br>430<br>663	454<br>480	NA	
**Low light control (before)**												
**wt**	80.7 ± 24.9	269.4 ± 78.2	4.9 ± 3.7	1.5 ± 1.0	42.1 ± 14.6	0	0	0	554.2 ± 207.5	13.1 ± 3.8	0.7 ± 0.2	966.7 ± 332.0
zep2 KO	69.5 ± 20.9	237.6 ± 75.1	3.3 ± 2.4	1.2 ± 0.8	34.5 ± 12.6	2.6 ± 1.2	0	0	492.4 ± 163.6	11.3 ± 4.0	0.5 ± 0.5	852.9 ± 276.2
zep2 KO2	68.2 ± 7.8	225.8 ± 27.2	4.4 ± 1.1	2.6 ± 0.2	27.0 ± 3.4	5.5 ± 1.3	0	0.1 ± 0.2	443.7 ± 56.7	10.9 ± 0.9	0.9 ± 0.1	789.0 ± 97.9
zep3 KO	85.2 ± 9.7	274.9 ± 41.1	7.1 ± 3.7	1.6 ± 0.2	40.2 ± 6.1	0	1.3 ± 0.4	0	588.1 ± 76.3	13.7 ± 1.3	0.5 ± 0.04	1012 ± 131
vde KO	84.8 ± 3.1	256.9 ± 15.6	10.3 ± 9.3	2.1 ± 1.3	45.7 ± 4.3	0	0	0	536.5 ± 32.0	16.4 ± 1.9	0.2 ± 0.3	979.8 ± 46.5
zep2 C	75.4 ± 6.9	268.0 ± 25.0	2.8 ± 2.0	1.6 ± 0.6	40.9 ± 4.1	0	0	0	538.7 ± 48.1	16.1 ± 1.8	0.8 ± 0.3	944.3 ± 88.2
zep3 C	94.2 ± 5.0	320.4 ± 1.9	4.6 ± 1.1	2.9 ± 0.3	35.0 ± 1.9	0	9.7 ± 0.9	0	635.5 ± 34.1	17.8 ± 1.5	0.3 ± 0.5	1120 ± 61.0
vde C	70.4 ± 4.7	238.8 ± 10.2	2.2 ± 1.4	2.8 ± 0.3	40.1 ± 1.8	0	0	0	498.0 ± 30.0	15.0 ± 1.1	0.5 ± 0.5	867.8 ± 48.8
**High light acclimated (5 days HL:LL)**												
**wt**	63.4 ± 4.3	205.4 ± 6.8	5.1 ± 1.3	2.7 ± 2.4	33.6 ± 1.1	0	0.3 ± 0.3	0	439.6 ± 52.1	11.9 ± 0.4	0.7 ± 0.4	762.5 ± 58.3
zep2 KO	63.2 ± 6.9	211.8 ± 14.2	3.6 ± 2.5	2.6 ± 0.8	31.0 ± 3.4	5.7 ± 0.3	0.9 ± 0.8	2.1 ± 1.5	450.6 ± 52.9	11.4 ± 0.7	0.3 ± 0.6	783.1 ± 72.0
zep2 KO2	NA	NA	NA	NA	NA	NA	NA	NA	NA	NA	NA	NA
zep3 KO	67.7 ± 7.3	211.7 25.4	5.1 ± 4.9	0.4 ± 0.3	14.3 ± 1.7	0	19.6 ± 2.3	0	456.1 ± 59.6	11.6 ± 2.0	0.2 ± 0.3	786.7 ± 93.3
vde KO	49.2 ± 1.4	146.2 ± 7.1	5.8 ± 5.7	0.4 ± 0.4	29.6 ± 3.3	0	0	0	351.6 ± 22.5	10.8 ± 1.4	0.6 ± 0.2	594.3 ± 29.6
zep2 C	NA	NA	NA	NA	NA	NA	NA	NA	NA	NA	NA	NA
zep3 C	61.2 ± 4.2	202.1 ± 16.6	3.8 ± 3.5	1.7 ± 0.6	24.6 ± 2.0	0	5.1 ± 0.7	0	436.2 ± 25.8	12.2 ± 0.7	0.4 ± 0.01	747.2 ± 46.8
vde C	43.6 ± 2.2	146.3 ± 4.6	4.5 ± 4.1	1.1 ± 0.4	24.2 ± 1.0	0	0	0	335.2 ± 19.0	9.9 ± 0.4	0.4 ± 0.2	565.1 ± 27.4

Pigment content is expressed as pmol:10^6 cells ratio (average ± sd, n=3; 0 = not detected; NA = not available). Representative retention time (rt) and λ_{max} in our HPLC system are reported. Pigments are displayed according to retention time: Chl c: chlorophyll *c*; Fx: fucoxanthin; Fx-i: fucoxanthin isomer; Vx: violaxanthin; Dd: diadinoxanthin; Ax: antheraxanthin; Dt: diatoxanthin; Zx: zeaxanthin; Chl a: chlorophyll *a*; β-car: β,β-carotene; c-β-car: cis-β,β-carotene. Gray background

highlights significant differences between the corresponding mutant line and wild type within each time point, according to adjusted p value of multiple comparison t-test ($p < 0.05$). The corresponding raw data is available as Supplementary Table S6.

Supplementary References

Bai, Y., Cao, T., Dautermann, O., Buschbeck, P., Cantrell, M.B., Chen, Y., Lein, C.D., Shi, X., Ware, M.A., Yang, F., Zhang, H., Zhang, L., Peers, G., Li, X., Lohr, M., 2022. Green diatom mutants reveal an intricate biosynthetic pathway of fucoxanthin. Proc. Natl. Acad. Sci. 119. https://doi.org/10.1073/pnas.2203708119

Blommaert, L., Chafai, L., Bailleul, B., 2021. The fine-tuning of NPQ in diatoms relies on the regulation of both xanthophyll cycle enzymes. Sci. Rep. 11, 1–16. https://doi.org/10.1038/s41598-021-91483-x

Bowler, C., Allen, A.E., Badger, J.H., Grimwood, J., Jabbari, K., Kuo, A., Maheswari, U., Martens, C., Maumus, F., Otillar, R.P., Rayko, E., Salamov, A., Vandepoele, K., Beszteri, B., Gruber, A., Heijde, M., Katinka, M., Mock, T., Valentin, K., Verret, F., Berges, J.A., Brownlee, C., Cadoret, J.-P., Chiovitti, A., Choi, C.J., Coesel, S., De Martino, A., Detter, J.C., Durkin, C., Falciatore, A., Fournet, J., Haruta, M., Huysman, M.J.J., Jenkins, B.D., Jiroutova, K., Jorgensen, R.E., Joubert, Y., Kaplan, A., Kröger, N., Kroth, P.G., La Roche, J., Lindquist, E., Lommer, M., Martin–Jézéquel, V., Lopez, P.J., Lucas, S., Mangogna, M., McGinnis, K., Medlin, L.K., Montsant, A., Secq, M.-P.O., Napoli, C., Obornik, M., Parker, M.S., Petit, J.-L., Porcel, B.M., Poulsen, N., Robison, M., Rychlewski, L., Rynearson, T.A., Schmutz, J., Shapiro, H., Siaut, M., Stanley, M., Sussman, M.R., Taylor, A.R., Vardi, A., Von Dassow, P., Vyverman, W., Willis, A., Wyrwicz, L.S., Rokhsar, D.S., Weissenbach, J., Armbrust, E.V., Green, B.R., Van De Peer, Y., Grigoriev, I.V., 2008. The *Phaeodactylum* genome reveals the evolutionary history of diatom genomes. Nature 456, 239–244. https://doi.org/10.1038/nature07410

Buck, J.M., Río Bártulos, C., Gruber, A., Kroth, P.G., 2018. Blasticidin-S deaminase, a new selection marker for genetic transformation of the diatom *Phaeodactylum tricornutum*. PeerJ 6, e5884. https://doi.org/10.7717/peerj.5884

Buck, J.M., Sherman, J., Bártulos, C.R., Serif, M., Halder, M., Henkel, J., Falciatore, A., Lavaud, J., Gorbunov, M.Y., Kroth, P.G., Falkowski, P.G., Lepetit, B., 2019. Lhcx proteins provide photoprotection via thermal dissipation of absorbed light in the diatom *Phaeodactylum tricornutum*. Nat. Commun. 10, 4167. https://doi.org/10.1038/s41467-019-12043-6

Bulankova, P., Sekulić, M., Jallet, D., Nef, C., Van Oosterhout, C., Delmont, T.O., Vercauteren, I., Osuna-Cruz, C.M., Vancaester, E., Mock, T., Sabbe, K., Daboussi, F., Bowler, C., Vyverman, W., Vandepoele, K., De Veylder, L., 2021. Mitotic recombination between homologous chromosomes drives genomic diversity in diatoms. Curr. Biol. 31, 3221-3232.e9. https://doi.org/10.1016/j.cub.2021.05.013

Gilmore, A.M., Mohanty, N., Yamamoto, H.Y., 1994. Epoxidation of zeaxanthin and antheraxanthin reverses non-photochemical quenching of photosystem II chlorophyll *a* fluorescence in the presence of trans-thylakoid ΔpH. FEBS Lett. 350, 271–274. https://doi.org/10.1016/0014-5793(94)00784-5

Goss, R., Ann Pinto, E., Wilhelm, C., Richter, M., 2006a. The importance of a highly active and ΔpH-regulated diatoxanthin epoxidase for the regulation of the PS II antenna function in diadinoxanthin cycle containing algae. J. Plant Physiol. 163, 1008–1021. https://doi.org/10.1016/j.jplph.2005.09.008

Goss, R., Böhme, K., Wilhelm, C., 1998. The xanthophyll cycle of *Mantoniella squamata* converts violaxanthin into antheraxanthin but not to zeaxanthin: consequences for the mechanism of enhanced non-photochemical energy dissipation. Planta 205, 613–621. https://doi.org/10.1007/s004250050364

Goss, R., Lepetit, B., Wilhelm, C., 2006b. Evidence for a rebinding of antheraxanthin to the light-harvesting complex during the epoxidation reaction of the violaxanthin cycle. J. Plant Physiol. 163, 585–590. https://doi.org/10.1016/j.jplph.2005.07.009

Nymark, M., Sharma, A., Hafskjold, M., Sparstad, T., Bones, A., Winge, P., 2017. CRISPR/Cas9 Gene Editing in the Marine Diatom *Phaeodactylum tricornutum*. BIO-Protoc. 7, 1–12. https://doi.org/10.21769/BioProtoc.2442

Nymark, M., Sharma, A.K., Sparstad, T., Bones, A.M., Winge, P., 2016. A CRISPR/Cas9 system adapted for gene editing in marine algae. Sci. Rep. 6, 24951. https://doi.org/10.1038/srep24951

Sharma, A.K., Nymark, M., Sparstad, T., Bones, A.M., Winge, P., 2018. Transgene-free genome editing in marine algae by bacterial conjugation – comparison with biolistic CRISPR/Cas9 transformation. Sci. Rep. 8, 1–11. https://doi.org/10.1038/s41598-018-32342-0

Siefermann, D., Yamamoto, H.Y., 1975. NADPH and oxygen-dependent epoxidation of zeaxanthin in isolated chloroplasts. Biochem. Biophys. Res. Commun. 62, 456–461. https://doi.org/10.1016/S0006-291X(75)80160-4
